# Arabidopsis Sucrose Synthase 3 (SUS3) regulates starch accumulation in guard cells at the end of day

**DOI:** 10.1080/15592324.2023.2171614

**Published:** 2023-02-12

**Authors:** Lucia Piro, Sabrina Flütsch, Diana Santelia

**Affiliations:** a Institute of Integrative Biology, ETH Zürich, Zürich, Switzerland; b Department of Biological Analyses and References, Swiss Federal Institute of Metrology METAS, Bern, Switzerland

**Keywords:** starch, stomatal movements, sucrose synthase, guard cells

## Abstract

Starch in the stomatal guard cells is largely synthesized using carbon precursors originating from sugars imported from the leaf mesophyll. Such heterotrophic nature of guard cell starch synthesis prompted us to investigate the role of cytosolic sucrose synthases (SUS) in this pathway. Out of the six members of the Arabidopsis *SUS* gene family, *SUS3* was the most highly expressed isoform in guard cells. The Arabidopsis *sus3* mutant displayed changes in guard cell starch contents comparable to the Wild Type (WT) up until 6 h into the day. After this time point, *sus3* guard cells surprisingly started to accumulate starch at very high rates, reaching the end of the day with significantly more starch than WT. Based on the phenotype of the *sus3* mutant, we suggest that in guard cells, SUS3 is involved in the regulation of carbon fluxes towards starch synthesis during the second half of the day. SUS3 may be part of a previously predicted guard cell futile cycle of metabolic reactions, in which sucrose is re-synthesized from UDP-glucose to avoid excessive starch synthesis toward the end of the day. This is in contrast to typical storage organs, in which cytosolic SUS is required to produce ADP-glucose for starch synthesis.

Stomata are microscopic pores on the leaf surface operating as gateways for CO_2_, O_2_ and water vapor. Stomata are bordered by specialized guard cells, which modulate the opening and closing of the stomatal pore through changes in their turgor pressure. Starch turnover in the guard cells is critically important for stomatal movements. Within the first hour of light, starch is degraded to promote rapid stomatal opening.^1,2^ Starch is then resynthesized during the rest of day, in coincidence with stomatal closing.^2^ Upon plant exposure to high CO_2_ concentrations, starch accumulation is required for efficient stomatal closure.^3^ For a long time, it has been hypothesized that organic osmolytes, such as sucrose and malate, are released from starch turnover during stomatal movements to contribute to the changes in guard cell turgor.^4,5^ More recent research revealed that starch turnover yields glucose to maintain the cytosolic sugar pool as a readily available source of osmolytes, energy and signaling molecules to drive fast stomatal movements.^1^

Guard cells have features of both autotrophic and heterotrophic tissues. Despite they have some photosynthetic activity, CO_2_ is assimilated mainly via phosphoenolpyruvate carboxylase, and mitochondria are the main source of ATP.^6,7^ The heterotrophic nature of guard cells is also reflected in the way they form starch. At the beginning of the day, when stomata are fully open, guard cell photosynthesis provides some precursors to fuel the plastidial phosphoglucose isomerase-phosphoglucose mutase reactions.^8^ The remainder of carbon precursors derives from sugars imported from the mesophyll via the plasma membrane sugar transport proteins 1 and 4.^1^ In the afternoon, when stomata tend to close as the plant becomes carbon-saturated, the combination of reduced guard cell photosynthesis, along with the need of removing organic metabolites, promotes starch accumulation from cytosolic sugars. Sugar transporters at the plastid membrane, namely the Glucose-6-Phosphate/Phosphate translocators (GPTs, which import G6P) and Glucose-1-Phosphate transporters (G1PTs, which import G1P) are necessary for starch synthesis in guard cells during the second half of day, essentially taking over the activity of the plastidial enzymes (Figure 1).^8,9^

One additional component of such uncanonical guard cell starch synthesis pathway may be the sucrose synthases (SUS). SUS are cytosolic, plasmamembrane- or cytoskeleton-associated enzymes, catalyzing the reversible cleavage of sucrose in UDP/ADP-glucose and fructose. In storage organs, such as potato tubers, maize kernels, or pea embryos, SUS hydrolytic activity controls sink strength by providing ADP-glucose for starch synthesis (Sun *et al*., 1992^[10]^; Zrenner *et al*., 1995; Déjardin *et al*., 1997^[11,12]^). SUS was previously implicated in the regulation of stomatal conductance when overexpressed in the guard cells of tobacco plants,^13,14^ but its role in guard cell starch metabolism is unknown.

**Figure 1.**
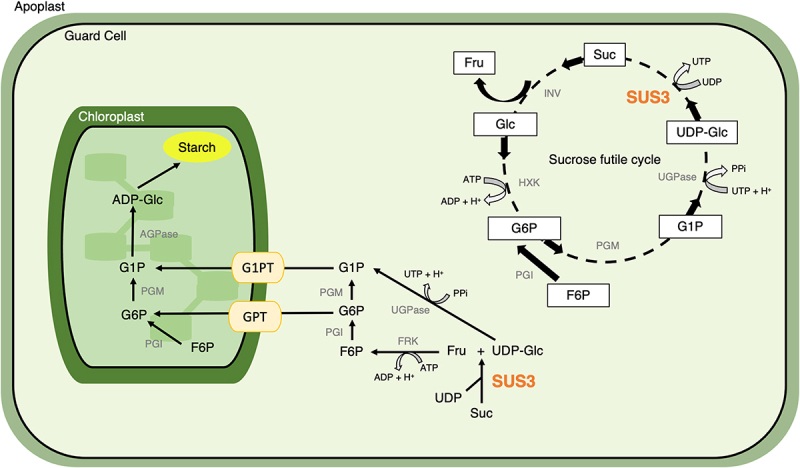
Predicted role of SUS3 in guard cell carbohydrate metabolism.

**Abbreviations**: Suc, sucrose; Fru, fructose; Glc, glucose; F6P, fructose-6-phosphate; G1P, glucose-1-phosphate; G6P, glucose-6-phosphate; UDP-Glc, uridine diphosphate-glucose; ADP-Glc, adenosine diphosphate-glucose; ATP, adenosine triphosphate; UTP, uridine triphosphate; INV, invertase; HKX, hexokinase; PGM, phosphoglucomutase; PGI, phosphoglucose isomerase; UGPase, UDPglucose pyrophosphorylase; AGPase, ADPglucose pyrophosphorylase; SUS3, sucrose synthase 3; FRK, fructokinase; GPT, G6P/phosphate translocator (GPT); G1PT, UDP-rhamnose/UDP-galactose transporters. Sucrose futile cycle modified from 6.

In Arabidopsis (*Arabidopsis thaliana*), SUS is a small gene family of six members, with each isoform generally showing a distinct expression profile, depending on the plant tissue, the developmental stage or the environmental conditions.^15,16^ We performed *in silico* gene expression analyses using the microarray data from Yang et al.^[17,18,19]^ In line with previous transcriptomic studies,^20,21^ we found that *SUS3* was the isoform most highly and preferentially expressed in guard cells, followed by *SUS1* (Figure 2a). We confirmed *SUS3* gene expression in guard cell-enriched epidermal peels by qPCR (Figure 2b). *SUS3* showed expression levels comparable with the guard cell-specific marker genes Myb transcription factor 60 (*MYB60*) and Inward-rectifying K^+^ channel 1 (*KAT1*). The expression of β -amylase 3 (*BAM3*; a leaf marker) was downregulated in guard cells, confirming the purity of the extracted guard cell-enriched epidermal peel samples. Based on the gene expression profile, we selected SUS3 for further investigations.

**Figure 2.**
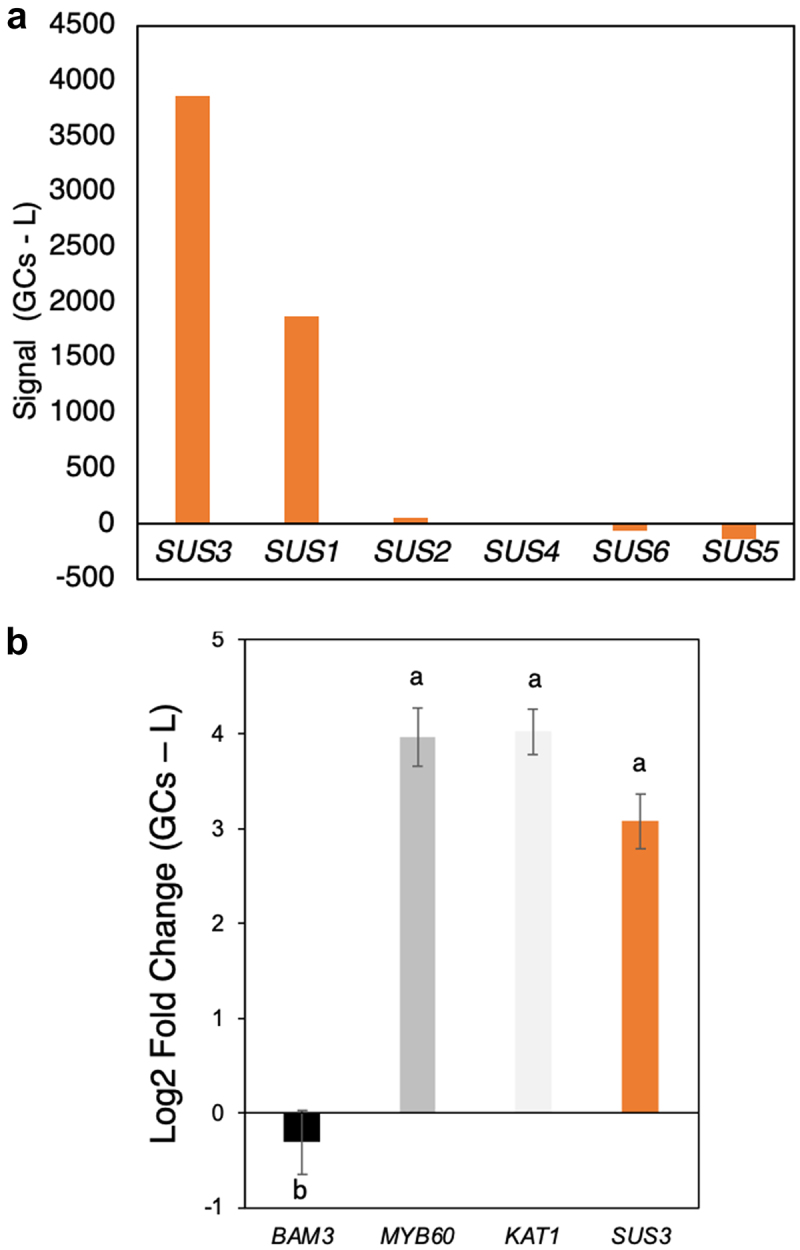
Gene expression analysis. (**a**) Relative gene expression analysis Guard Cells (GCs) minus Leaves (L) based on the published Micro Array data from Yang et al. (2008)^[17]^. (**b**)*SUS3* (*At4g02280*) expression in guard cell-enriched samples minus intact rosette at the end of the night. *BAM3* (*At4g17090*) was used as leaf-specific marker, while *MYB60* (*At1g08810*) and *KAT1* (*At5g46240*) as guard cell-specific markers. Two independent experiments where performed, n = 6. Means ± fold change is showed and *ACT2* (At3g18780) was used as housekeeping gene. ANOVA with post hoc Tukey’s test was performed (p = 0.05). Letters indicate significant statistical difference between the expression profile of the different genes. The qPCR primers used for the study were: *SUS3*-fwd GACCAGACTGATGAGCATGTCG; *SUS3*-rev TCTTCACTTTGTCGAGCCTCG; *BAM3, MYB60, KAT1* and *ACT2* primers as in Flütsch et al. (2022)[1].

We obtained a homozygous Arabidopsis T-DNA insertion line at the *SUS3* locus (*sus3*; SALK-019405) and examined changes in stomatal starch levels during the light phase (under a 12-h/12-h light/dark photoperiod). The *sus3* mutant displayed guard cell starch turnover comparable to the wild type (WT) up until 3 h of light, starting with similar amounts of starch at the end of the night (EoN) (Figure 3a). Consistent with these results, stomatal opening kinetics and photosynthetic assimilation rate upon transition from dark-to-light at dawn were unaltered in the *sus3* mutant (Figure 4).

**Figure 3.**
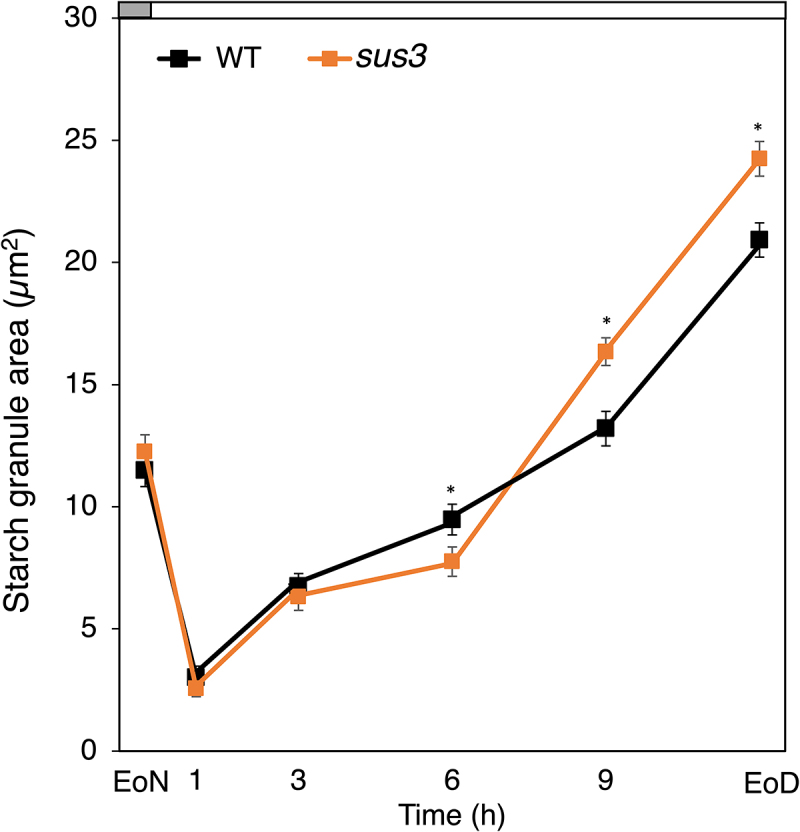
Guard cell starch dynamics. (**a**) Starch dynamics in guard cells of intact leaves of WT and *sus3* plants during the light phase. The gray bar on top of the graph represents the period of darkness, while the white bar the period of light. Plants were grown under 150 μmol m^–2^ s^–1^ of white light under a 12-h/12-h light/dark photoperiod with a temperature of 21°C/19°C and relative humidity (RH) of 45%/55%. Experiments were performed using 4-week-old plants according to ^14^. Data from three independent experiments are shown; means ± SEM; n = 120 individual guard cells per genotype and time point. Two tailed Student *t*-Test was performed per each time point (alfa = 0.05). The stars indicate significant difference between the two genotypes.

**Figure 4.**
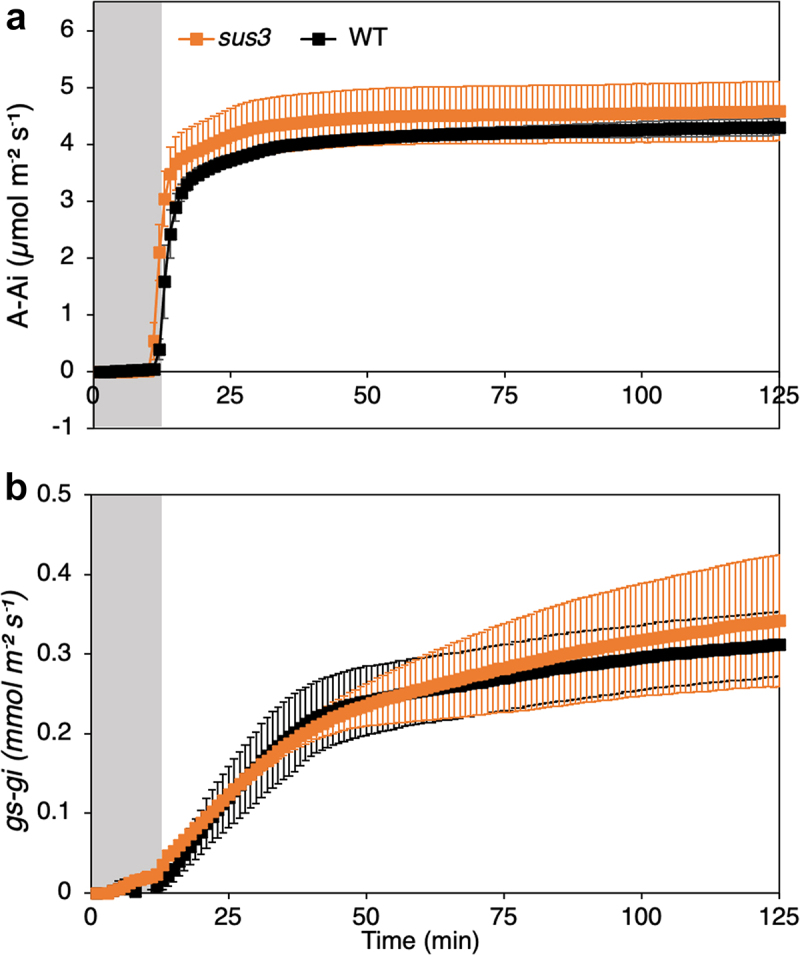
Photosynthetic rate and stomatal conductance. Normalized whole-plant recordings of (a) changes in CO_2_ assimilation(*A*) normalized by subtracting the initial *A* value (*A_i_*), and (**b**) stomatal conductance *g_s_* of WT and *sus3* plants in response to a shift from dark to light, normalized by subtracting the initial *g_s_* value (*g_si_*). Values were normalized to the values at the end of the night (T0). Plants were grown under a short-day condition 8-h/16-h light/dark at 150 μmol m^–2^ s^–1^ of white light. Temperature was maintained at 21°C/19°C while RH at 45%/55%. Measurements were done with 5 to 6-week-old plants. Shown is means ± SEM; n = 3 per genotype. No statistically significant differences were observed. Prior normalization, the values for the *g_s_* of the three WT biological replicates were recorded between 1.51 and 3.47 mmol m⁻^2^ s⁻^1^ while the *A* was between −1.82 and 3.46 µmol m⁻^2^ s⁻^1^. For *sus3,* the *g_s_* was between 0.54 and 1.95 47 mmol m⁻^2^ s⁻^1^ while the *A* was between −1.36 and 3.37 µmol m⁻^2^ s⁻^1^.

Between 3 and 6 h of light, however, starch accumulation was slightly, but significantly reduced in *sus3* guard cells compared to WT (Figure 3b). This suggests that a fraction of SUS3 degradation products may be directed toward starch synthesis at this time of day. It is reasonable to hypothesize that conversion of UDP-glucose and fructose to hexose sugars is followed by import into the plastid by GPT/G1PT transporters (Figure 1). In any event, the minimal reduction in starch amounts seen in the *sus3* mutant leads to the conclusion that most of SUS end-products are further metabolized to fuel ATP production via the tricarboxylic acid (TCA) cycle and oxidative phosphorylation in mitochondria, as previously suggested.^14^

Later during the day, between 6 h and 9 h of light, *sus3* guard cells surprisingly started to accumulate starch at very high rates (Figure 3b), reaching the end of the day (EoD) with significantly more starch than WT (Figure 3a). This unexpected phenotype can be explained by the predicted incidence in guard cells of the so-called “sucrose futile cycle” (Figure 1). The sucrose futile cycle is a hypothesized flux of carbons composed of five reactions where both synthesis and degradation of sucrose take place simultaneously.^6^ It was suggested that the sucrose futile cycle leads to the resynthesis of sucrose from UDP-glucose through the activity of SUS and it has the role of avoiding excessive starch synthesis, while maintaining the pool of cytosolic sugars in a readily available form to fuel glycolysis and mitochondrial metabolism according the need of the cell.^22^ In the light of these observations, it is likely that the over accumulation of starch in *sus3* guard cells results from the disruption of the sucrose futile cycle and subsequent redirection to starch of intermediate metabolites, such as G1P, via the activity of the plastidial G1PT transporters (Figure 1).^1^

To conclude, our study suggests that SUS3 represents a central node in the guard cell metabolism network, particularly towards the end of the day, when the guard cells have abundant amounts of sugars. By controlling the flux of carbon toward starch synthesis, SUS3 coordinates the need of energy with the removal of metabolites during stomatal closure at the end of the day. Future efforts should consider modulating SUS3 activity in guard cells to optimize stomatal movements and potentially increase plant water use efficiency and productivity.
